# Detection of Known and Novel Virus Sequences in the Black Soldier Fly and Expression of Host Antiviral Pathways

**DOI:** 10.3390/v16081219

**Published:** 2024-07-30

**Authors:** Hunter K. Walt, Heather R. Jordan, Florencia Meyer, Federico G. Hoffmann

**Affiliations:** 1 Department of Biochemistry, Nutrition and Health Promotion, Mississippi State University, Mississippi State, MS 39762, USA; hkw59@msstate.edu (H.K.W.); florencia.meyer@msstate.edu (F.M.); 2 Department of Biological Sciences, Mississippi State University, Mississippi State, MS 39762, USA; jordan@biology.msstate.edu; 3 Institute for Genomics, Biocomputing and Biotechnology, Mississippi State University, Mississippi State, MS 39762, USA

**Keywords:** virome, *Sigmavirus*, *Totivirus*, *Hermetia illucens*, virus Surveillance

## Abstract

The mass rearing of animals in close quarters can be highly conducive to microbe transmission, including pathogens. This has been shown multiple times in the case of important industrial insects such as crickets, silkworms, and honeybees. One industrial insect of increasing importance is the black soldier fly (Diptera: *Hermetia illucens*), as it can convert organic waste into high-quality protein and fatty acids. Along with this, they take up far less space than traditional protein sources, as millions of black soldier flies can be reared in a relatively small facility. Because of this, there is a growing interest in the pathogens that could impact black soldier fly-rearing efforts. So far, only three black soldier fly-associated viruses have been identified. We used metatranscriptomic sequencing to survey black soldier fly guts, frass, and diet for viruses. We detected sequences from two novel viruses. One, which we name *Hermetia illucens* sigma-like virus 1, is phylogenetically related to viruses of the genus *Sigmavirus*, which have been highly studied in *Drosophila*. The other novel virus, which we name *Hermetia illucens* inse-like virus 1, is the second double-stranded RNA virus of the order *Ghabrivirales* described in the black soldier fly, and groups within a new family of insect viruses called the *Inseviridae*. We also detected two black soldier fly-associated viruses previously identified by our group: BSF nairo-like virus 1 and BSF uncharacterized bunyavirus-like 1. Consistent with our previous study, these two viruses are found primarily in frass samples and occur together more often than expected at random. When analyzing host transcription, we found significant differences in gene expression for eight candidate antiviral genes in the black soldier fly when comparing samples with and without viral sequences. Our results suggest that black soldier fly–virus interactions are ongoing, and they could be of interest to black soldier fly producers.

## 1. Introduction

Insects provide an attractive alternative to traditional protein sources as a more sustainable and environmentally friendly option. The black soldier fly (Diptera: *Hermetia illucens*) (BSF) is a promising candidate as an alternative source of nutrition for livestock, pets, and humans [1,2]. This is mainly due to the ability of the BSF to convert organic waste into high quality proteins and fatty acids. Another appeal of the BSF as an alternative protein source is that millions of BSFs can be reared in a relatively small amount of space. The mass rearing of animals creates an opportune environment for the spread of microbes throughout a population. In the case of pathogens, this can be detrimental to an industry, and the production of other insects of industrial importance has been affected by viral epidemics in the past [3].

Until recently, there was not a manifested interest in the viruses harbored by the BSF. This is partially because BSFs are believed to have a highly robust immune system due to the fact that the BSF larval stage lives in compost, and their genome encodes some of the highest numbers of antimicrobial peptide (AMP) genes in insects [4,5,6]. So far, two studies have detected viruses in BSFs using shotgun metatranscriptomic approaches; they described three viruses associated with the BSF, one toti-like virus and two bunyaviruses [7,8]. Although the fitness effects of these viruses on BSFs have not been assessed, the presence of many endogenous viral elements (EVEs) in the BSF genome suggests that BSF has historically been infected by viruses [8].

Because the BSF virome is poorly characterized and interest in BSF production is increasing worldwide, we used a shotgun metatranscriptomic approach to detect viruses associated with BSFs. We surveyed larval gut (*n* = 36), frass (*n* = 36), and diet (*n* = 4) transcriptome samples (*n* = 76 total) and detected two known and two novel BSF-associated viruses. Interestingly, one of the novel viruses is related to a *Drosophila* virus that has been routinely studied as a host–pathogen coevolution model in *Drosophila*. The other novel virus groups within a family of insect-infecting double-stranded RNA (dsRNA) viruses called the *Inseviridae*, which the International Committee on Taxonomy of Viruses (ICTV) recently ratified (https://ictv.global/taxonomy/, accessed on 17 July 2024) [9]. These viruses could be of interest to BSF producers, as they may affect BSF fitness. Finally, we found that multiple BSF candidate antiviral genes were differentially expressed in samples where viral sequences were detected.

## 2. Materials and Methods

### 2.1. BSF and Substrate Sampling

BSF larvae were sampled from an experiment determining the performance of BSF larvae reared on four different diet substrates of varying nutritional profiles. Briefly, larvae were reared in 50 mL conical plastic tubes and fed either a Gainesville diet (industry standard), a protein-biased diet (1C:5P), a carbohydrate-biased diet (5C:1P), or a balanced diet (1C:1P). After the feed trial, larvae and frass samples were stored in a freezer at −80 °C. We sampled nine larvae from each of the four diets (*n* = 36) and briefly submerged them in 70% ethanol followed by a sterile water rinse. We dissected whole larval guts in RNAlater solution (Invitrogen, Waltham, MA, USA) in a sterile Petri dish using a SteREO Discovery microscope (Zeiss, Oberkochen, Germany) and immediately placed them in 1.5 mL of RNAzol (Molecular Research Center, Cincinnati, OH, USA) for RNA isolation. Concordantly, we sampled 1 g of substrate (frass) from each enclosure and placed it in 1.5 mL of RNAzol for RNA isolation. Samples of diet without larvae were run alongside the feed trial study, so we also sampled 1 g of diet from each diet-only enclosure (*n* = 4) and placed it into 1.5 mL RNAzol for RNA isolation.

### 2.2. RNA Isolation

All samples were homogenized in RNAzol using a Genolyte 1200 tissue homogenizer (Spex SamplePrep, Metuchen, NJ, USA) at 4000 RPM for one minute using sterile 3 mm stainless steel balls. The resulting homogenate was spun down for five minutes at 12,000× *g*, and 1 mL of the supernatant was used for RNA isolation. The manufacturer’s protocol for RNA isolation using RNAzol was followed through DNA, protein, and polysaccharide precipitation, upon which the aqueous fraction containing the RNA was directly placed into a NEB Monarch RNA cleanup kit (New England Biolabs, Ipswich, MA, USA), also following the manufacturer’s protocol. RNA purity was measured using a NanoDrop One spectrophotometer (Thermo Fisher Scientific, Grand Island, NY, USA), and RNA concentration was assessed using a Qubit 2.0 fluorometer (Invitrogen, Waltham, MA, USA). RNA integrity was assessed using a 4150 TapeStation System (Agilent, Santa Clara, CA, USA).

### 2.3. Library Preparation and Sequencing

Whole shotgun metatranscriptome sequencing libraries were prepared using NEB Ultra II RNA kits, but rRNA depletion and mRNA enrichment steps were avoided to keep all host and microbial reads. The resulting cDNA was multiplexed using NEBNext Oligos for Illumina (New England Biolabs, Ipswich, MA, USA). The resulting libraries were pooled by diet, and the quality of the library was assessed using a 4150 TapeStation System (Agilent, Santa Clara, CA, USA). The resulting libraries were sequenced on a HiSeq 2000 instrument (Illumina, San Diego, CA, USA), generating 151 base pair reads.

### 2.4. Metatranscriptome Assembly

The quality of the reads was assessed using fastQC v.0.11.9 [10], and adapters and low-quality bases were trimmed using Trimmomatic v.0.39 [11]. Black soldier fly reads were discarded from further analyses by mapping all sequencing datasets to the black soldier fly reference genome (GCF_905115235.1) [12] using HISAT2 v.2.2.1 [13] with default parameters and redirecting the unmapped reads to a new file using the –un-conc-gz option. For each dataset, the unmapped reads were assembled using Trinity v.2.14.0 through the Trinity docker container [14,15]. All transcripts were uniquely named across every transcriptome, and all transcriptomes were concatenated into one file. Only transcripts greater than 500 nucleotides were retained, and these were clustered at a minimum sequence identity of 90% using cd-hit-est with a word size of eight [16].

### 2.5. Identification of Virus Sequences

All clustered transcripts were searched against the NCBI nr database using DIAMOND in the BLASTX mode employing the –very-sensitive option with an e-value cutoff of 1 × 10^−2^ [17,18]. All sequences with alignments to known RNA viruses were further inspected for the presence of open reading frames using NCBI’s orffinder tool (https://www.ncbi.nlm.nih.gov/orffinder/, accessed on 5 May 2023), and the resulting proteins were analyzed for conserved protein domains using NCBI’s conserved domain database search tool (https://www.ncbi.nlm.nih.gov/Structure/cdd/wrpsb.cgi, accessed on 5 May 2023) and the InterProScan web server [19].

### 2.6. Phylogenetic Analysis of Novel Virus Sequences

To assess the identity of potentially novel viruses, we conducted phylogenetic analyses based on viral RNA-dependent RNA polymerase (RDRP). We collected a diverse set of viral RDRP sequences based on predicted viral order from conserved protein domain searches, and BLASTX searches (https://blast.ncbi.nlm.nih.gov/Blast.cgi, accessed on 5 May 2023) against NCBI’s non-redundant protein database. We aligned the amino acid sequences using the g-ins-i algorithm in MAFFT v.7.490 [20]. To build the phylogenetic trees, we used IQ-TREE v.2.0.7 using ModelFinder to identify the best-fitting model [21,22,23]. Branch support was evaluated using the Shimodaira–Hasegawa-like approximate likelihood ratio test (SH-aLRT) with 1000 replicates, the aBayes test, and the ultrafast bootstrap algorithm with 1000 replicates [24,25,26,27]. The resulting phylogenetic trees were viewed and annotated using the Interactive Tree of Life web server [28]. For the tree including *Hermetia illucens* inse-like virus 1, we initially used a large selection of sequences representing all families within the order *Ghabrivirales* to infer our phylogeny (Figure S1). After mid-point rooting of the tree, we selected the most recent highly supported node (SH-alrt ≥ 80%, abayes ≥ 0.9, and ≥95% UF bootstrap) coalescing from *Hermetia illucens* inse-like virus 1, *Hermetia illucens* toti-like virus 1, and an outgroup and used these sequences to generate a new alignment and phylogeny using the methods described earlier in this section. P-distance calculations were performed using MEGA v.11.0.13 [29].

### 2.7. Overlap of Virus Occurrence across Samples

To test if the amount of co-occurrence in viruses across samples was more than one would expect at random, we used Fisher’s Exact Test implemented in R [30].

### 2.8. Quantification of BSF and Viral Transcripts

To determine if viral infection influenced putative antiviral gene expression in BSF, we pseudoaligned the trimmed read datasets from larval samples to the RefSeq BSF transcriptome using Salmon v0.14.1, using the reference genome as a decoy (as described here: https://combine-lab.github.io/alevin-tutorial/2019/selective-alignment/, accessed on 21 February 2024) [31]. After mapping, we imported the abundance estimates from Salmon to DESeq2 [32] using tximport [33]. One sample was discarded due to a low number of reads (gcl9_b). The read counts of the remaining samples were normalized between samples using the DESeq command in DESeq2, and the resulting normalized count matrix was used for all subsequent analyses.

To quantify viral transcripts, we mapped the trimmed read datasets to the longest transcripts from *Hermetia illucens* inse-like virus 1 and *Hermetia illucens* sigma-like virus 1 using Kallisto v0.44.0 [34]. We did not map to BSF uncharacterized bunyavirus-like 1 or BSF nairo-like virus 1, as these viruses were primarily detected in frass samples, and thus non-contiguous reads present in larval samples could be the result of contamination instead of viral infection in the BSF gut. We considered viruses “present” in a sample when more than 10 reads mapped to either virus transcript.

### 2.9. Identifying Candidate Antiviral Genes in BSF

To identify candidate antiviral genes, we conducted a literature search to find relevant antiviral genes in *Drosophila* and mosquitoes [35]. We used these sequences to find orthologous genes present in the BSF genome using best reciprocal blast hits between *Drosophila melanogaster* or *Aedes aegypti* proteins. Along with this, we used eggNOG mapper v.2.1.9 in DIAMOND mode to assign KEGG identifiers to BSF genes putatively belonging to the major antiviral/immune pathways Toll and Imd (map04624), JAK/STAT (map04630), RNAi (K11593), and piRNA (K02156). Finally, we used the sequences of BSF AMPs and lysozymes identified by Vogel et al. (2018) to BLAST against the BSF genome and obtained the gene names for all putative AMPs/lysozyme genes in BSF. Using these genes, we tested for significant differences in putative antiviral BSF gene expression using the normalized read counts from DEseq2 as well as the Wilcoxon rank-sum test implemented in R. Genes with mean normalized counts less than 10 across all samples were not considered.

## 3. Results

**Figure 1:**
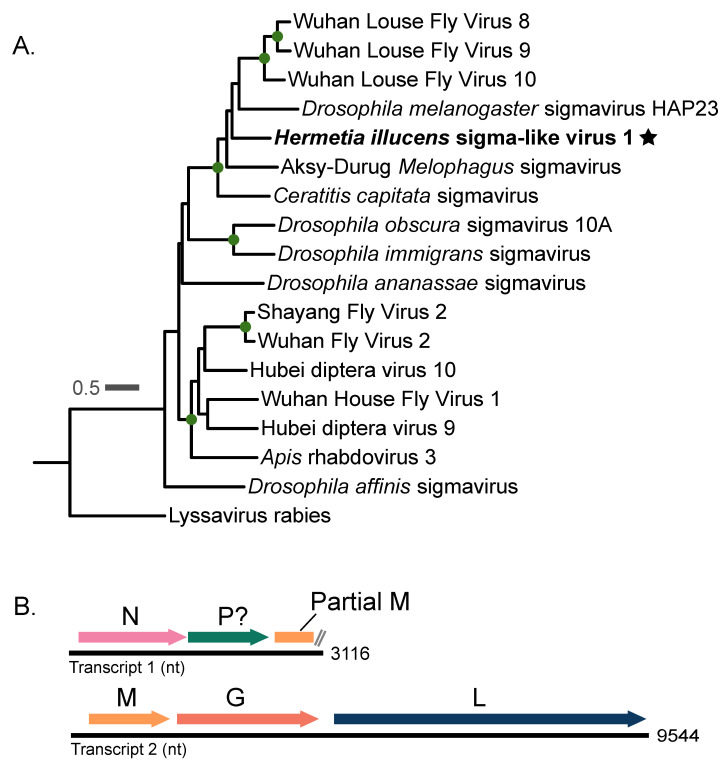
*Sigmavirus* phylogeny and genomic structure of *Hermetia illucens* sigma-like virus 1. (**A**) Phylogeny of the sigmaviruses based on an alignment the RDRP protein, including *Hermetia illucens* sigma-like virus 1, which is denoted by a star. Green dots along the branches represent highly supported nodes: SH-alrt ≥ 80%, abayes ≥ 0.9, and ≥95% UF bootstrap. Another virus from the family *rhabdoviridae*, lyssavirus rabies, was used as the outgroup. The phylogeny was inferred using the LG+F+I+G4 substitution model. (**B**) Genomic structure of *Hermetia illucens* sigma-like virus 1 according to our study in positive-sense polarity. The question mark next to the P gene denotes that we could not confirm homology by sequence similarity. The fragmented genome is probably a result of metatranscriptomic assembly error, as no other sigmaviruses are multipartite.

**Figure 2:**
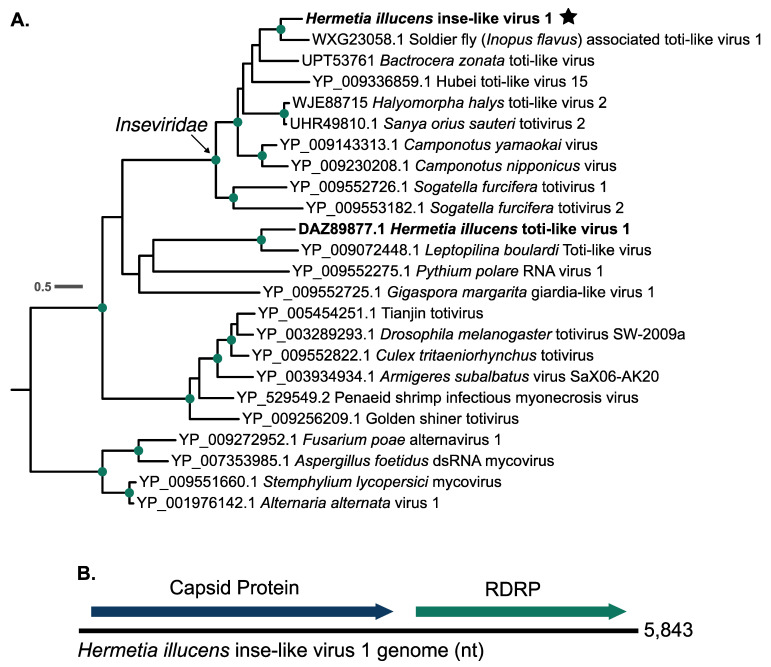
*Hermetia illucens* inse-like virus 1 groups within a highly supported clade of viruses within the family *Inseviridae*. (**A**) RDRP amino acid alignment of a selection of viruses of the order *Ghabrivirales* (see Section 2.6 and Figure S1). Green dots along the branches represent highly supported nodes: SH-alrt ≥ 80%, abayes ≥ 0.9, and ≥95% UF bootstrap. BSF viruses are highlighted in bold, and BSF viruses discovered in this study are denoted by a star. The tree was generated using the LG+F+R4 substitution model and rooted at the branch leading to the clade of fungal viruses at the bottom of the tree. (**B**) Genome structure of *Hermetia illucens* inse-like virus 1.

Our 76 initial individual metatranscriptome assemblies resulted in 4,315,370 total transcripts. After filtering and clustering our metatranscriptome assemblies, the total number of transcripts was reduced to 381,196. Using BLASTX, we detected sequences from two known BSF-associated viruses [7] and two novel virus sequences in our metatranscriptomic assemblies (Figure 1 and Figure 2).

### 3.1. Hermetia illucens Sigma-like Virus 1

**Figure 3:**
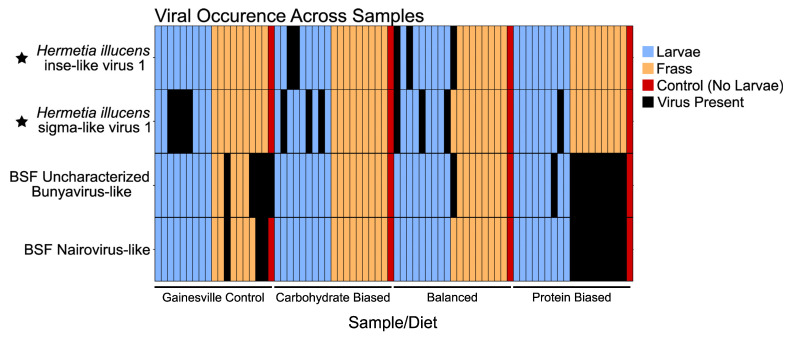
Occurrence of viruses across all samples used for this study. Viruses were counted as “present” in a sample if a transcript (>500 nt) clustered (90% sequence similarity) with a viral transcript from CD-hit clustering. The novel viruses detected in this study are denoted by a star. BSF uncharacterized bunyavirus-like 1 and BSF nairo-like virus 1 occur together more often than expected at random (Fisher’s Exact Test: *p* = 5.867 × 10^−11^).

We detected two novel virus sequences in our study. One sequence had multiple BLAST alignments (E-value = 0) to proteins belonging to the sigmaviruses. From now on, we refer to this sequence as *Hermetia illucens* sigma-like virus 1. To investigate the phylogenetic relationships of this virus to other sigmaviruses, we aligned the RDRP of *Hermetia illucens* sigma-like virus 1 to a selection of sigmavirus RDRP sequences from RefSeq along with its top five closest BLAST hits. We used Rabies virus as an outgroup based off a previous study showing that it branches sister to the sigmaviruses in the rhabdovirus phylogenetic tree [36]. Our phylogeny shows that *Hermetia illucens* sigma-like virus 1 groups within a clade of dipteran-infecting sigmaviruses, including a Drosophila melanogaster sigmavirus and multiple louse fly-infecting sigmaviruses (Figure 1A). Between the RDRP proteins used in our sigmavirus phylogeny, the mean p-distance is 0.56. The virus with the lowest p-distance to *Hermetia illucens* sigma-like virus 1 is Wuhan Louse Fly Virus 10, with a p-distance of 0.451 in the RDRP region. All pairwise p-distances for the RDRPs used in the sigmavirus phylogeny (Figure 1A) are shown in Supplementary Data 1. The genome of *Hermetia illucens* sigma-like virus 1 was fractionated across two transcripts, one 3116 nt transcript containing the sigmavirus nucleocapsid protein gene (N), the putative polymerase-associated protein gene (P), and a partial matrix protein (M), and another 9544 nt transcript containing three other canonical sigmavirus genes: the complete M protein gene, the spike protein (G) gene, and the RDRP-containing L gene (Figure 1B). *Hermetia illucens* sigma-like virus 1 was detected in 11 samples from our study, all of which were larval gut samples (Figure 3, Table S1).

### 3.2. Hermetia illucens Inse-like Virus 1

Along with *Hermetia illucens* sigma-like virus 1, we assembled a transcript that had significant BLAST alignments to dsRNA viruses of the order *Ghabrivirales*. Interestingly, the closest BLAST hits to this transcript are sequences from Soldier fly-associated toti-like virus 1 (GenBank: PP410011), a virus that was discovered in the sugar cane soldier fly [37]. The viral transcript we detected and Soldier fly-associated toti-like virus 1 share a 50.77% nucleotide identity between their entire genomes and 48.07% amino acid identity between their RDRP proteins. To understand the phylogenetic relationship of this novel virus sequence to others, we aligned its translated RDRP region of with a large set of RDRP amino acid sequences from viruses within the *Ghabrivirales* to determine its closest phylogenetic relatives (Figure S1). We used a subset of these sequences for further analyses (see Section 2.6). We found that the novel virus sequence we detected groups in a highly supported clade of insect-infecting toti-like viruses, which have recently been assigned to a new viral family called the *Inseviridae* by the ICTV in release MSL39 (https://ictv.global/taxonomy/taxondetails?taxnode_id=202315168&taxon_name=Inseviridae, accessed on 24 June 2024) (Figure 2A) [9]. We hereon refer to this sequence as Hermetia illucens inse-like virus 1. Hermetia illucens inse-like virus 1 branches sister to soldier fly associated toti-like virus 1 (Figure 2A). Within the *Inseviridae* clade in our phylogeny, the mean p-distance is 0.60 between the RDRP proteins. For the whole phylogeny, the mean p-distance is 0.78 between the RDRP proteins. Pairwise p-distances for the RDRP proteins used in Figure 2A are shown in Supplementary Data 1. The *Hermetia illucens* inse-like virus 1 genome is a 5843 bp containing two large ORFs: one encoding a capsid protein, and one encoding the RDRP protein (Figure 2B). *Hermetia illucens* inse-like virus 1 was found in five total samples, four deriving from larval gut metatranscriptomes and one deriving from a frass metatranscriptome (Figure 3, Table S1).

### 3.3. Known BSF-Associated Viruses

We detected multiple transcripts with substantial homology (BLASTx e-value = 0, ~70–99% aa identity in RDRP) to two previously discovered virus sequences associated with BSF: BSF Uncharacterized bunyavirus-like 1 and BSF nairo-like virus 1. These viruses were primarily detected in frass samples, similar to when we identified them [7] (Figure 3, Table S1). We confirmed that these sequences phylogenetically group with the original sequences we presented in our last study (Figure S2) [7]. BSF uncharacterized bunyavirus-like 1 occurred in 16 samples, with only one being derived from the BSF larval gut (Figure 3, Table S1). BSF nairo-like virus 1 was detected in 12 samples, all of which were frass samples (Figure 3, Table S1). Interestingly, these two viruses occur very regularly together. Every time BSF Nairo-like virus 1 is detected, it co-occurs with BSF uncharacterized bunyavirus-like 1, resulting in them occurring together 12 out of 16 times when one is present. This is significantly more than one would expect at random (Fisher’s Exact Test, *p* = 5.867 × 10^−11^).

### 3.4. Antiviral Gene Expression in BSF

**Figure 4:**
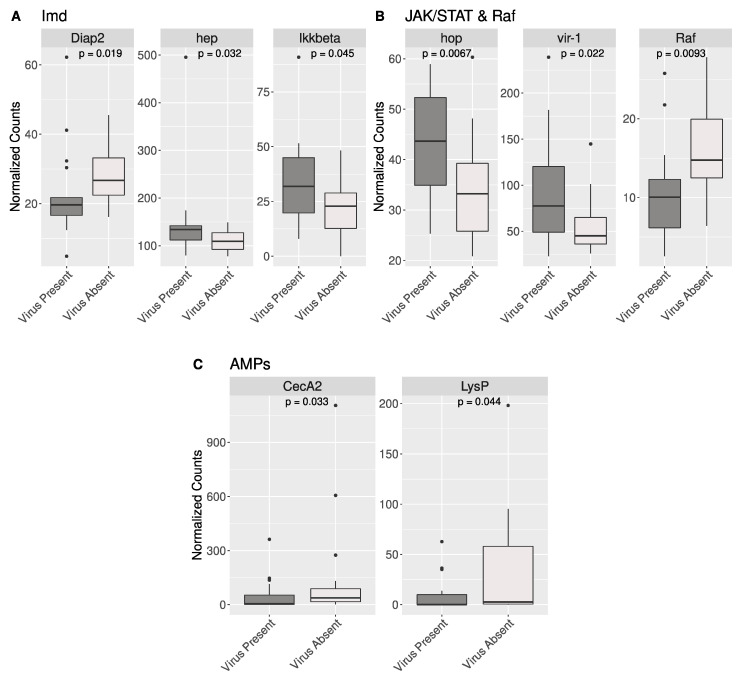
Eight candidate antiviral genes have significant differences in gene expression between virus-present BSF samples and virus-absent BSF samples. (**A**) Three genes putatively from the Imd pathway in BSF had significant differences in gene expression in virus-present vs. virus-absent BSF. Diap2 was upregulated in virus-absent samples, while hep and IKKbeta were upregulated in virus-present BSF samples. (**B**) Two genes from the JAK/STAT pathway were upregulated in virus-present BSF samples. Along with these, Raf, a gene whose product has known interactions with the JAK/STAT pathway, was upregulated in virus-absent BSF samples. (**C**) Two AMPs were significantly upregulated in virus-absent samples. All gene names are derived from their ortholog in *Drosophila melanogaster*.

We identified 210 candidate antiviral genes from the dipteran immune/antiviral pathways Toll, Imd, JAK/STAT, RNAi, and piRNA using KEGG or a literature search and BLAST (Supplementary Data 2). We tested all these genes for significant differences in expression between BSF samples that were classified as “virus-present” (greater than 10 reads mapped to a virus genome) relative to samples classified as “virus absent” (less than 10 reads mapped to a virus genome). We found eight genes with significant differences in expression: three BSF orthologs associated with the Imd pathway in other dipteran insects, three associated with the JAK/STAT pathway in other dipterans, and two BSF AMPs (Figure 4). These genes along with the function of their Drosophila orthologs are shown in Table 1.

## 4. Discussion

### 4.1. Hermetia illucens Sigma-like Virus 1

Our study identifies two novel BSF-associated viruses and two previously described BSF-associated viruses. Before this, only three exogenous viruses had been found in association with BSF [7,8]. One of the viruses discovered in this study, which we call *Hermetia illucens* sigma-like virus 1, is phylogenetically related to the sigmaviruses (Figure 1). *Sigmavirus* is a genus of negative-sense single-stranded RNA viruses within the family *Rhabdoviridae* that mostly infect insects of the order Diptera [36,38,39,40]. Generally, sigmaviruses have a monopartite genome of around 12 kb to 15 kb (https://ictv.global/report/chapter/rhabdoviridae/rhabdoviridae/sigmavirus, accessed on 19 February 2024) [9]. In our study, the *Hermetia illucens* sigma-like virus 1 genome is split into two different transcripts (Figure 1B). In metatranscriptomic studies, it is not unusual to find fragments of viral genomes, which could be due to the processing of the samples or errors introduced by the complexity of metatranscriptome assembly. All canonical sigmavirus genes were present across the two detected transcripts (Figure 1B), although we could not confirm that a large ORF on transcript 1 was the P gene based on sequence similarity, as it had no significant BLAST hits to other viruses or conserved domains. However, we propose that this is the P gene due to its position between the N gene and a partial M gene on this transcript (Figure 1B).

Sigmaviruses are used to study host–pathogen coevolution in the model organism *Drosophila melanogaster* [41,42,43,44]. *Drosophila* sigmaviruses are vertically transmitted by both males and females, and while *Drosophila* sigmaviruses are not known to be lethal to the host, they could be of interest to BSF breeders, as they are associated with negative fitness effects such as delayed development and decreased fecundity [44,45]. However, not all effects of viral infection may be negative. Interestingly, one study found that sigmavirus infection in *Drosophila* positively influenced male reproductive success [46].

### 4.2. Hermetia illucens Inse-like Virus 1

Another novel virus detected in this study, which we call *Hermetia illucens* inse-like virus 1, groups within a clade of insect-infecting viruses formerly classified within the family *Totiviridae*, but recent revisions in the ICTV taxonomy have placed them in a new family called the *Inseviridae* [9,47,48]. *Inseviridae* is comprised of insect-infecting dsRNA viruses that were formerly referred to as totiviruses or toti-like viruses, although viruses of the family *Totiviridae* generally infect fungi and protists. More recently, many metatranscriptomic studies have detected toti-like viruses in insects, including BSF [8,48,49,50,51,52,53,54]. In our phylogeny, *Hermetia illucens* inse-like virus 1 groups within a highly supported clade that includes several of the exemplary insevirus species according to the ICTV taxonomy release MSL39 (https://ictv.global/news/taxonomy_2023, accessed on 24 June 2024) [9] and groups sister to a virus that was isolated from a different species of soldier fly (Figure 2A). *Hermetia illucens* inse-like virus 1 is the second dsRNA virus of the order *Ghabrivirales* discovered in the BSF [8]. The first one, *Hermetia illucens* toti-like virus 1, falls in a separate clade that is sister to the *Inseviridae* and branches with a virus detected in a species of parasitoid wasp [55] (Figure 2A). Although our phylogeny places *Hermetia illucens* toti-like virus 1 in a clade sister to the *Inseviridae*, they are highly diverged from each other, with a p-distance of 0.8362 between their RDRP proteins (Supplementary Data 1).

### 4.3. Known BSF Viruses Detected in Our Study

The two known viruses that we detected in this study occurred together very frequently, which is consistent with what we observed in our previous study [7]. These viruses were mostly detected in frass samples (Figure 3), which is also consistent with our previous study. Interestingly, a portion of BSF uncharacterized bunyavirus-like 1 genome was detected in a sample where no larvae were present, posing the question of whether BSF is the true host of this virus or if it infects a component of their diet. Bunyaviruses can infect a wide variety of species, but our previous study showed that BSF uncharacterized bunyavirus-like 1 was most closely related to insect-infecting viruses [7]. Alternatively, this could be the result of cross-contamination of samples, as only partially assembled sequences (2438 nt and 1261 nt instead of ~5800 nt) were found in this sample.

### 4.4. Gene Expression in Putative Antiviral Genes in BSF

Although metatranscriptomics is useful for virus discovery and surveillance, it does not provide any information about the effects of viral infection on the host. To understand the effects that viruses may have on BSF, we analyzed the host transcriptome of BSF samples that were classified as either “virus present” or “virus absent”. From a compiled table of 210 candidate genes (Supplementary Data 2) involved in BSF response to viruses, eight of them were differentially expressed. Of these, six are associated with the signaling pathways Imd and JAK/STAT (Figure 4), both of which can trigger antiviral immune responses [56,57]. One of the BSF genes that is significantly upregulated in virus-positive samples is orthologous to vir-1, a gene regulated by the JAK/Stat pathway, which is also upregulated in *Drosophila* when infected by viruses (Figure 4, Table 1) [57,58]. Interestingly, we did not find significant differences in expression between orthologous components of the RNAi and PIWI-interacting RNA pathways (piRNA), both of which are important antiviral pathways in various dipteran species [35]. However, our sequencing datasets did not allow for the detection of small RNAs, the central molecules in the recognition of foreign nucleic acid. Evaluating the small RNA repertoire of BSF could provide more insight into the activation of antiviral pathways in this system [59]. Finally, we acknowledge that this method is exploratory, and factors other than the presence of viruses could influence the expression of these genes.

## 5. Conclusions

Understanding the viruses routinely associated with the BSF could be helpful in the event of a viral epidemic, which could cause detrimental impacts to the BSF industry. Our metatranscriptomic approach can detect known and novel viruses in the BSF and frass and emphasizes the value of metatranscriptomic-based techniques to survey the viruses in the BSF. Our approach also allowed the identification of host genes that could potentially be exploited as a screening tool for infection or fitness. However, functional studies are still necessary to understand the pathogenicity of these viruses in the BSF.

## Figures and Tables

**Table 1:** Significantly differentially expressed BSF genes and the function of their *Drosophila melanogaster* orthologs. Genes that are downregulated in virus-present samples are shown in blue text, while genes that are upregulated in virus-present samples are in red text.

BSF Gene	Drosophila Ortholog	Function in Drosophila
LOC119654977	Diap2	Mediator of NF-kB signaling—required for innate immune response
LOC119653405	hep	Critical for JNK activation in immune signaling
LOC119646668	IKKbeta	Regulates antiviral response in Imd
LOC119651484	hop	Induces expression of JAK/STAT regulated genes
LOC119646570	vir-1	Regulated by JAK/STAT; expressed in response to viral infection
LOC119656159	Raf	Component of Ras/Raf pathway; interacts with hop of JAK/STAT
LOC119653270	CecA2	Antimicrobial peptide
LOC119654763	LysP	Antimicrobial activity against Gram-negative bacteria

## Data Availability

The RNA-seq reads generated in this study were deposited in NCBI’s sequence read archive under BioProject ID: PRJNA1128605, and representative *Hermetia illucens* sigma-like virus 1 and *Hermetia illucens* inse-like virus 1 genomes were submitted to GenBank under the accessions PP968021–PP968023.

## Supplementary Materials

The following supporting information can be downloaded at: https://www.mdpi.com/article/10.3390/v16081219/s1, Supplementary Data 1: P-distance matrices between the RDRP sequences used for the phylogenies in Figure 1A and Figure 2A. Supplementary Data 2: List of candidate antiviral BSF gene IDs, the predicted ortholog assigned by eggNOG (or *Drosophila* ortholog), the predicted antiviral pathway it belongs to, the tool used to determine orthology, and the source where we identified the gene as potentially antiviral (i.e., homology searches or literature searches). Figure S1: Large phylogeny of *Ghabrivirales* sequences, with shaded areas indicating the sequences used for Figure 2A. Figure S2: Phylogenetic confirmation of known BSF-associated viruses. Table S1: Summary Table of Figure 3.
