# Supplementary material for: Detection of Known and Novel Virus Sequences in the Black Soldier Fly and Expression of Host Antiviral Pathways
Source: Viruses. 2024 Jul 30;16(8):1219. doi: 10.3390/v16081219 (PMC11359925; doi:10.3390/v16081219)
Supplement: Supplementary file 1 [file viruses-16-01219-s001.zip › supplementary_tables_and_figures.pdf]

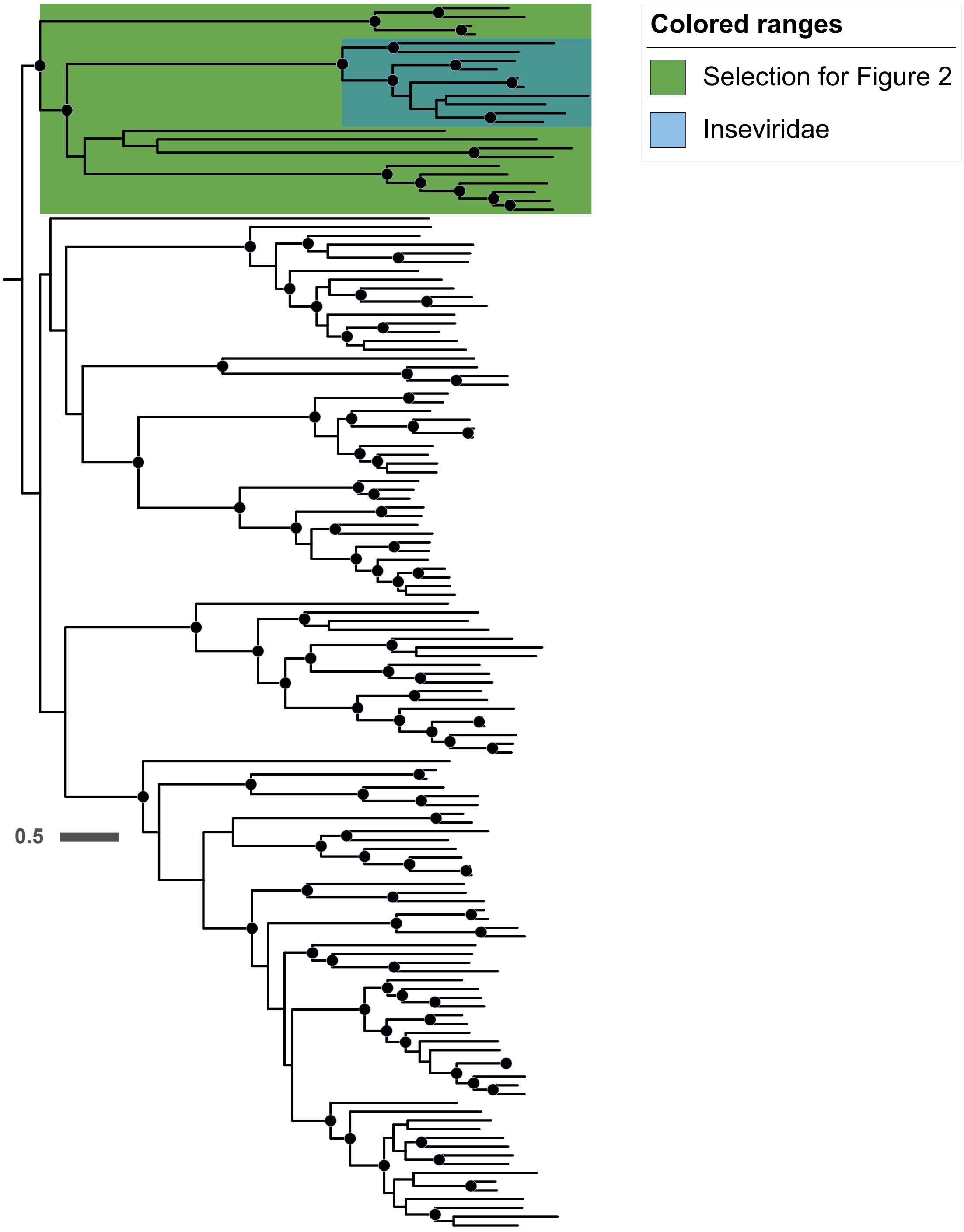

Figure S1: Full *Ghabrivirales* RDRP phylogeny. Shaded areas represent the selection of viral RDRP sequences used to generate the phylogeny in Figure 2A. Dots along the branches represent highly supported nodes (SH-alrt ≥ 80%, abayes ≥ 0.9, and ≥ 95% UF bootstrap). The tree was generated using the LG+F+R8 substitution model and midpoint rooted.

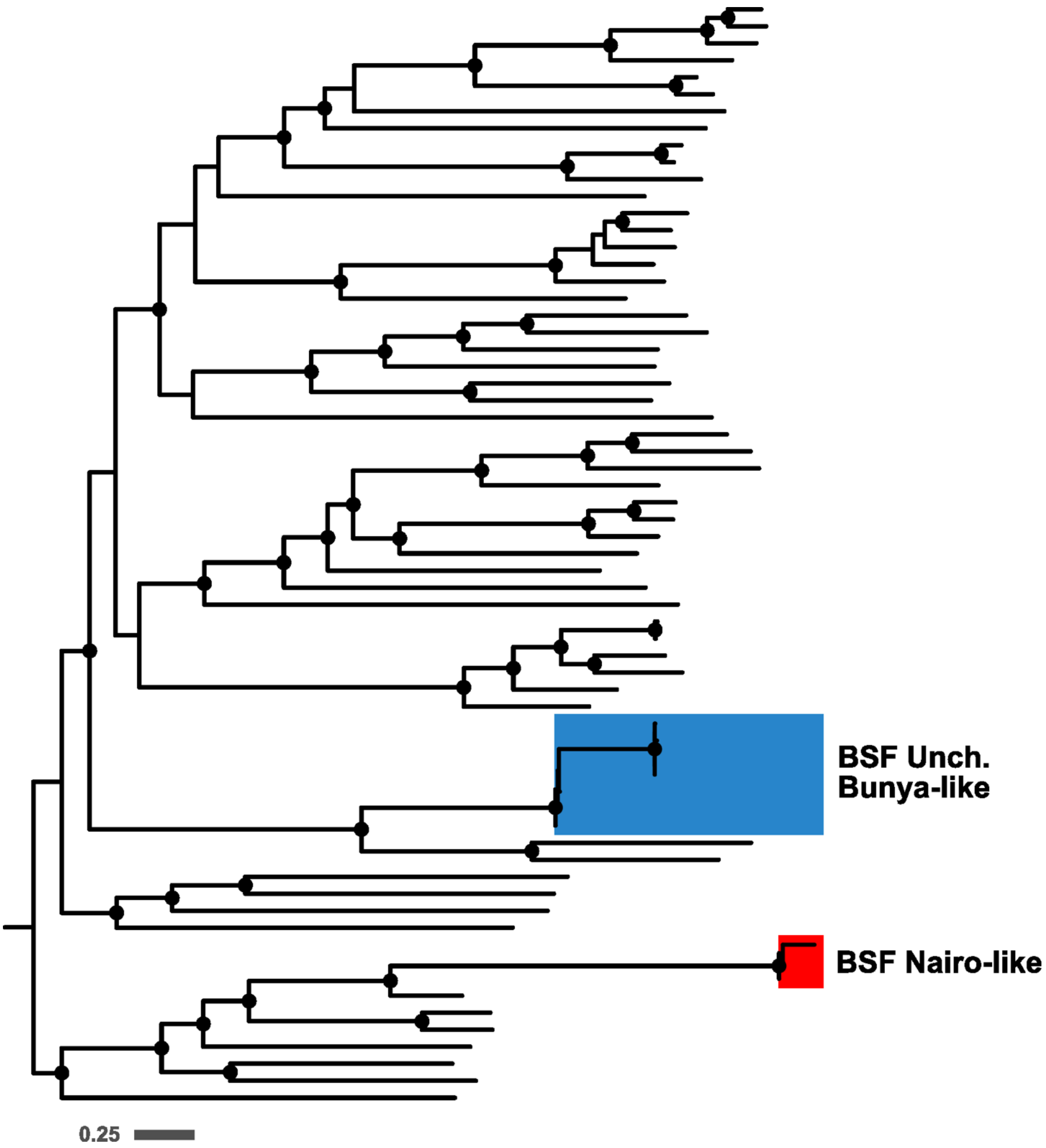

Figure S2: Phylogenetic confirmation of known BSF viruses detected in our study. The Phylogeny was built using RDRP sequences and the same taxanomic selection of the *Bunyavirales* from Walt et. al (2023), Figure 1. We added the representative sequences (CD-hit) from each known BSF virus detected in this study. The known BSF viruses form highly supported clades with the original sequences discovered in Walt et al. (2023), although there are some diverged sequences in both clades. Dots along the tree indicate highly supported nodes (SH-alrt ≥ 80%, abayes ≥ 0.9, and ≥ 95% UF bootstrap). The tree was generated using the VT+F+R7 substitution model and midpoint rooted.

Table S1: Summary of virus occurrence across samples. HiIv 1 = *Hermetia illucens* inse-like virus 1, HiSv 1 = *Hermetia illucens* sigma-like virus 1. BSF Unc 1 = BSF Uncharacterized bunya-like virus 1. BSF Nairo 1 = BSF Nairo-like virus 1. This Table is visually represented in Figure 3 of the main text.

	Gainesville Diet			Carbohydrate Biased Diet			Balanced Diet			Protein Biased Diet		
	Larvae	Frass	Diet	Larvae	Frass	Diet	Larvae	Frass	Diet	Larvae	Frass	Diet
HiIv 1	0	0	0	2	0	0	2	1	0	0	0	0
HiSv 1	4	0	0	3	0	0	3	0	0	1	0	0
BSF Unc 1	0	4	1	0	0	0	0	1	0	1	9	0
BSF Nairo 1	0	3	0	0	0	0	0	0	0	0	9	0
